# A Novel System to Increase Yield of Manufacturing Test of an RF Transceiver through Application of Machine Learning

**DOI:** 10.3390/s23020705

**Published:** 2023-01-08

**Authors:** Atif Siddiqui, Pablo Otero, Muhammad Zubair

**Affiliations:** 1 Airbus Defence and Space, UK; 2 Telecommunications Engineering School, University of Malaga, 29010 Málaga, Spain; 3 Institute of Oceanic Engineering Research, University of Malaga, 29010 Málaga, Spain; 4 Faculty of Engineering, Iqra University, Karachi 75850, Pakistan

**Keywords:** RF testing, automated test equipment, machine learning, LabVIEW, yield, boundary scan

## Abstract

Electronic manufacturing and design companies maintain test sites for a range of products. These products are designed according to the end-user requirements. The end user requirement, then, determines which of the proof of design and manufacturing tests are needed. Test sites are designed to carry out two things, i.e., proof of design and manufacturing tests. The team responsible for designing test sites considers several parameters like deployment cost, test time, test coverage, etc. In this study, an automated test site using a supervised machine learning algorithm for testing an ultra-high frequency (UHF) transceiver is presented. The test site is designed in three steps. Firstly, an initial manual test site is designed. Secondly, the manual design is upgraded into a fully automated test site. And finally supervised machine learning is applied to the automated design to further enhance the capability. The manual test site setup is required to streamline the test sequence and validate the control and measurements taken from the test equipment and unit under test (UUT) performance. The manual test results showed a high test time, and some inconsistencies were observed when the test operator was required to change component values to tune the UUT. There was also a sudden increase in the UUT quantities and so, to cater for this, the test site is upgraded to an automated test site while the issue of inconsistencies is resolved through the application of machine learning. The automated test site significantly reduced test time per UUT. To support the test operator in selecting the correct component value the first time, a supervised machine learning algorithm is applied. The results show an overall improvement in terms of reduced test time, increased consistency, and improved quality through automation and machine learning.

## 1. Introduction

An RF electronic product goes through several stages during design and manufacturing. The focus of this research is the different types of tests a product must undergo during design and manufacturing. The testing during the product design is referred to as the proof of design test or verification and validation test. These tests vary depending on the type of RF electronic product. The next phase is the design for manufacturing (DFM) and design for testability (DFT). A new RF electronic product is ready for manufacturing after the successful conclusion of all proof of design tests, DFM, and DFT stages. Then comes the manufacturing process which is made up of several stages including the manufacturing test. 

**Figure 1:**
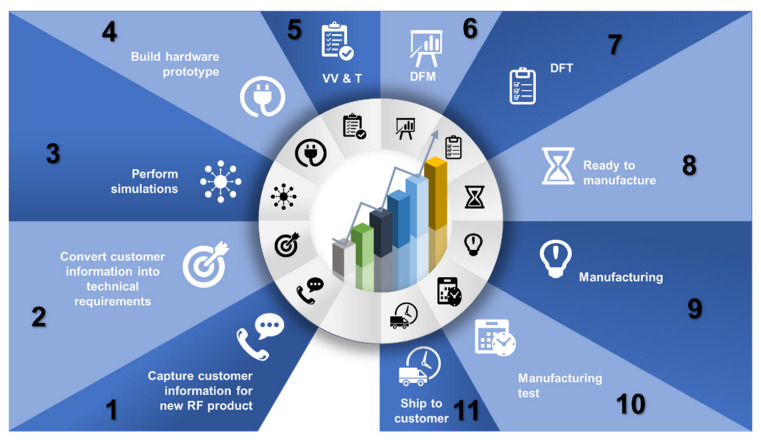
RF electronic product design and manufacturing process life cycle.

Figure 1 shows the RF electronic product lifecycle which includes all design and manufacturing activities. A new RF electronic product goes through proof of design tests which are defined based on the customer requirements. Successful completion of proof of design tests kick-starts the DFM and DFT processes. Both DFM and DFT processes can go through a few iterations before the product becomes ready for manufacturing. The actual manufacturing process for the RF product is dependent on several parameters including customer requirements, budget, unit under test (UUT) batch size, time to market, etc. 

Typical steps of the manufacturing process are shown in Figure 1. The manufacturing process starts with the fabrication of the Printed Circuit Board (PCB). The PCB is then populated with components which can be surface mount technology (SMT) components or through-hole components. Depending on the type and complexity of the product, the PCB can be multi-layered and can have components that can be soldered on the top side, bottom side, or both. Once the PCB is populated with components, it is then referred to as a printed circuit board assembly (PCBA). The PCBA then goes through a visual inspection. The next step is testing the UUT which in this case is the PCBA. 

The end-users prefer good quality products at a lower cost. The manufacturing companies, in order to meet customer requirements, focus on manufacturing quality products at a low cost with a quick turnaround. Test operators are required to take decisions at certain stages of the process which means there are inconsistencies. This increases both the test time and time to market. In this manuscript machine learning is applied to solve this problem and a specific scenario is selected to demonstrate the effectiveness of machine learning in order to increase yield and improve consistency.

The study is presented as follows. Section 2 covers the related work which includes details of some RF electronic products followed by a review of some existing RF and other electronic products test sites and details of different types of tests that are performed on RF electronic products. Finally, a review of machine learning algorithms and their applications concludes this section. The novelty of this research work is presented in Section 3. The four steps of the research methodology are discussed in Section 4. The proposed test site details are in Section 5. The next Section 6 list various activities related to the implementation and validation of the proposed test site. The overall discussion is in Section 7 followed by the conclusion and future work in Section 8.

## 2. Related Work

**Figure 2:**
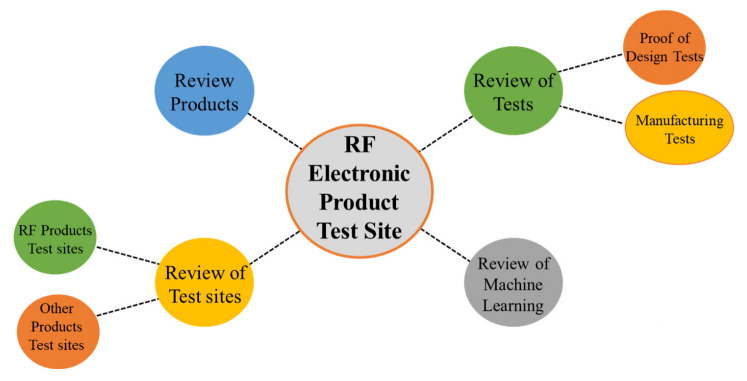
Literature review map.

Figure 2 shows the scope of the literature review. This includes four categories of reviews: the product review, the test site review, the test review, and the machine learning review. 

### 2.1. RF Electronic Products Review

A review is carried out to collect data for different RF products and their variants. The data collection technique is discussed in [1]. RF product types are determined by the operating frequency which indicates how these products can be tested. A list of some RF products is presented in Table 1.

### 2.2. Existing RF and Other Electronic Products Test Sites Review

The cost of RF electronic products test sites can vary depending on the operating frequency. Some of the RF test sites also include tuning options where the UUT is required to be modified to cater for this feature. In this section some RF test sites are reviewed.

In [2] authors presented an architecture for testing IoT-based systems. The key to designing a good test site is to employ design for testability tools used to setup an optimized test site [3]. Continuity testing to detect short/open circuit is a useful test for the validation of RF PCBs [4]. In a typical test setup, depending on the requirements, the first test stage is usually a Bed of Nails (BoN) based test [5]. The authors in [6] designed a test site that includes a troubleshooting feature. The importance of an ATE system is discussed in [7].

A remotely controlled test site via the Internet is presented in [8] where the authors used LabVIEW [9] for implementation. The authors used a built-in interface for controlling laboratory test equipment [10]. This type of control is a common feature in RF test sites. Some RF products require high-speed data capture and processing, and the authors in [11] used a LabVIEW-based DAQ system to achieve this. In [12,13], a typical RF test site is implemented where a network analyzer is deployed for taking measurements. When setting up test sites it is important to consider the performance parameters, some of which are discussed in [14]. LabVIEW is a popular software tool used in setting up test sites for RF products. The use of LabVIEW in testing the space system is presented in [15]. The authors in [16] demonstrated a scenario where the standard laboratory test equipment is remotely controlled. In [17] a standalone PXI-based test site is used for testing RF electronic circuits. Some RF test sites are setup to acquire and store analog waveforms. 

The authors in [18] used an oscilloscope for waveform acquisition. In [19] an automated test site is used for the unit under test. RF electronic products also require a separate test site for testing antennas. The authors discussed a test site for antenna testing using a commercially available control board [20]. A test site for testing PCBs is presented in [21]. A GPIB-based test site is presented in [22]. The software-defined radio test site is published by authors in [23]. Microcontroller-based control boards are also used in acquiring low-voltage signals as discussed in [24]. A complex RF test site is presented in [25] where the device under test is a configurable device and the tests are focused on finding tuning faults. Faulty cables are one of the main reasons for product failure. A cable test site is also reviewed in [26]. An RF device under test is presented in [27]. The authors’ setup automated testing for the units under test in [28,29]. 

The review presented in this section is summarized and presented in Table 2. 

### 2.3. RF Electronic Products Test Categories

RF electronic products go through two different tests i.e., the proof of design (PoD) test and the manufacturing test. Some RF products also include a Built-In Self-Test (BIST) which provides a further level of validation. A technique to design test sites for electronic products and how tests can be setup are discussed in [31].

#### 2.3.1. Verification and Validation Test

This is also called a proof of design test and includes several tests carried out during the RF electronic product design phase. These tests are used to confirm that the electronic product design is as per the specifications. Some of the proof of design tests are presented in this section.

- A drop test is used to determine the integrity of the UUT after going through a physical impact. The main considerations are the height and angle from where the UUT is dropped. The UUT for this test is normally fitted inside the casing.
- Accelerated Life Test (ALT) determines the performance of the electronic product throughout its end of life. In this test, the UUT goes through a hot and cold temperature cycle for several weeks. Some key measurements are taken during this test to record the UUT performance.
- A vibration Test is carried out on the UUT assembly i.e., fitted inside the casing. The things to consider are the vibration profile and a mechanical mount that is used to fit the UUT on the vibration equipment.

#### 2.3.2. Manufacturing Test

Manufacturing tests are selected based on customer requirements and UUT specifications. Some of the manufacturing tests are listed in this section.

- Open layer testing (UUT is not powered) is performed on the PCBs offline i.e., the power supply is not connected. Some of the tests are done using the JTAG port and the rest of the tests include checking in-circuit component values.
- Open layer testing (UUT is powered) is a functional test where the UUT is tested for both manufacturing faults and confirmation of some design parameters. The functional testing can be semi-automated where the test software guides the operator to probe various test points on the UUT. The test software then takes measurements through test equipment and records test results. The functional test can also be fully automated where the test equipment picks up the test signals from UUT using a Bed of Nails (BoN) jig.
- Cable testing is also an important test for RF electronic products. The performance of the cables used in these products varies based on the operating frequency and impedance. These cables and harnesses are both tested independently as well as part of the UUT assembly.

### 2.4. Machine Learning Techniques

The application of machine learning in setting up test sites and testing electronic products is discussed in this section. 

The authors reviewed supervised and unsupervised machine learning algorithms in [32,33] and presented their findings. Like other systems and processes, there are some limitations of machine learning, some of which are highlighted in [34,35]. The application of machine learning in setting up test sites for electronic products and analysis is presented in [36]. The application of machine learning to review and improve quality is discussed in [37]. A python-based toolkit is used by authors in [38]. The authors used machine learning to process test data collected during manufacturing [39]. System performance is predicted using machine learning [40].

## 3. The Novelty of the Proposed Research Work

In this manuscript, a test site is designed for an ultra-high frequency transceiver through three steps. In the first step, a manual test site is designed for the UUT. This process highlights the limitation, delays, and errors and provides an opportunity for operator training. The test site connectivity and performance are also validated through a manual process. The main limitation of the test site is the high test time where consistency and repeatability are also highlighted. The high test time is reduced through automation where the test equipment is controlled, the test follows a fixed sequence, and test results are stored. The test software is developed in LabVIEW. UUT quality is improved after automation as repeatability and consistency are achieved. 

During the test, the UUT is required to be tuned by changing some component values to bring the measurements within the limits. This is a critical test stage where the operator is required to select component values, solder on the printed circuit board, take measurements again, and repeat this until the results are within the limits. This process increases the test time as well as reduces consistency even after automation. 

**Figure 3:**
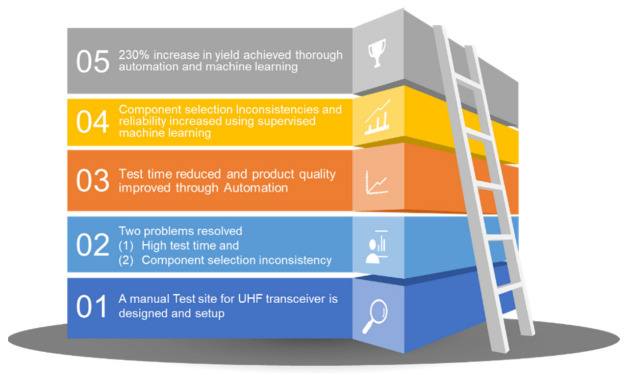
Novel features of the proposed process.

To solve this issue, a machine learning algorithm is applied which takes the measurement as an input. Using the historical data, the algorithm suggests the best component values to the operator. This feature reduces inconsistencies and test time significantly. In the end, a 230% increased yield is achieved through reduced test time, improved quality, and increased consistency. The novel features of this test site are shown in Figure 3. 

## 4. Research Methodology

**Figure 4:**
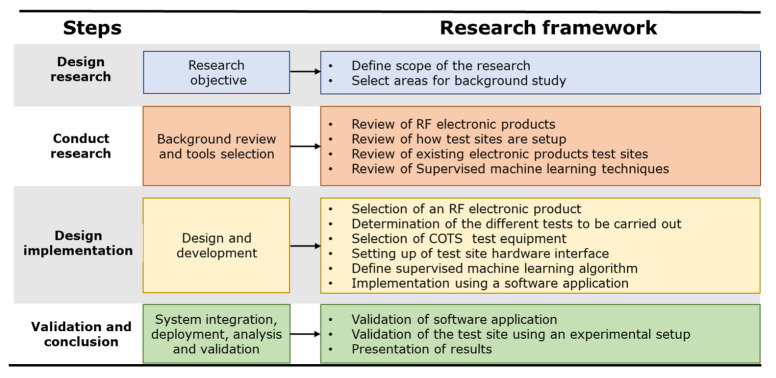
Research methodology.

This research is a step towards defining a process to test RF electronic products. Figure 4 highlights the four steps of the research methodology.

### 4.1. Design Research

The research is initiated by identifying some categories which include the identification of different RF products, how existing test sites are setup, what type of testing is performed and finally a review of machine learning to be applied to RF product testing. 

### 4.2. Research Steps

The first step within this category is to identify different types of RF products and review their features. This is important as this activity will determine what type of testing is needed and how the test sites can be designed. The details are listed in Section 2.1.

The next step is to review the existing test sites designed and what features are implemented. The focus is on both software and hardware tools used. The details are listed in Section 2.2. 

In the third step, the capabilities of test sites are reviewed to determine what tests can be carried out. Section 2.3 lists some of the tests that are done during the product lifecycle from design to manufacturing. Some of these tests are performed during the design phase while others are performed during the manufacturing of the product.

The last step in this category is focused on machine learning. Some machine learning techniques are reviewed. This is followed by the implementation of machine learning to solve different problems. Some researchers used existing toolkits to implement machine learning while others highlighted some limitations or advantages. Section 2.4 includes the details of this review. 

### 4.3. Design Implementation 

The first step is to select a product that will be reviewed and then a test site is setup for this product. In this step, a UHF transceiver is selected as the UUT.

The transceiver is reviewed, and a number of tests are selected which are carried out during the manufacturing process of this product. The tests are selected keeping in mind the test coverage. The tests also cover the main portions of the product to make sure that no design or manufacturing faults exist. 

In the third step different test equipment are reviewed. The review is based on the cost, frequency range, and accuracy of the test equipment.

The fourth step is to set up the test site hardware. In this step, the test equipment is connected with each other and to the UUT. Cables and harnesses are carefully selected to cope with the frequency and other requirements to keep external interference to a minimum. Once everything is setup, a manual test is carried out to make sure the test site is operational.

In the last two steps, a machine learning algorithm is implemented using a software application. This test software also includes controlling the test equipment, taking measurements, storing results, and performing analysis.

### 4.4. Validation and Conclusions

The final step is to validate the experimental setup. This is done by first validating the test software. The testing is carried out on the UHF transceiver and finally the results are presented, stored, and analyzed. The machine learning algorithm is also used to provide information to the test engineer to rectify the faults.

## 5. Proposed Machine-Learning-Based Automated System

### 5.1. System Block Diagram

**Figure 5:**
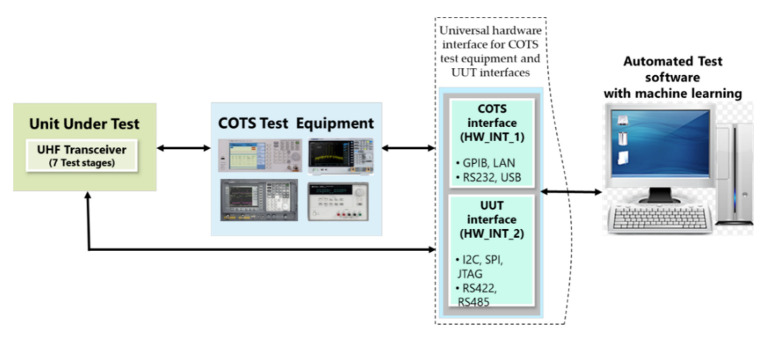
Machine learning-based UHF product test site block diagram.

**Figure 6:**
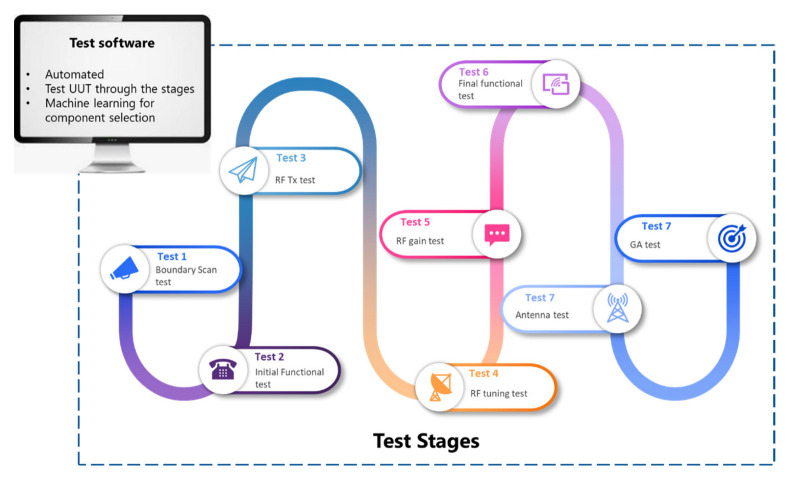
Machine learning-based UHF product test site test stages.

The system block diagram is presented in Figure 5. Test results generated through each test stage, as shown in Figure 6, are stored on the main server. The test equipment required for each test stage is shown on the block diagram. The test site is setup in such a way that the test equipment can be used and moved between test stages. The test site for the UHF transceiver is implemented using a universal hardware interface module as presented in [39]. The interface module provides a link between the test software and test equipment. The test site is designed using the figure of merit parameters discussed in [36].

### 5.2. Database

A database is setup for this test site which includes details of existing test equipment and an option to add new test equipment. The structure of the database is presented in [36]. 

### 5.3. Machine Learning Algorithm

This research is the continuation of the work published in [41], where a standard approach is defined for testing electronic products based on a figure of merit and machine learning. The same approach is extended and applied to an RF electronic product and results are presented. The research work is based on real data which is also used for validation through experimental setup. A good-quality and low-cost product is something each customer is looking for. As with other areas, there are lot of errors generated and consistency is reduced due to human interactions at certain instances in the manufacturing test process, and machine learning help can resolve these issues. Through this manuscript a scenario is selected, a unique dataset for supervised machine learning is created and machine learning algorithm is then applied. The application of machine learning reduces the human error, increased quality and consistency and improved yield.

**Figure 7:**
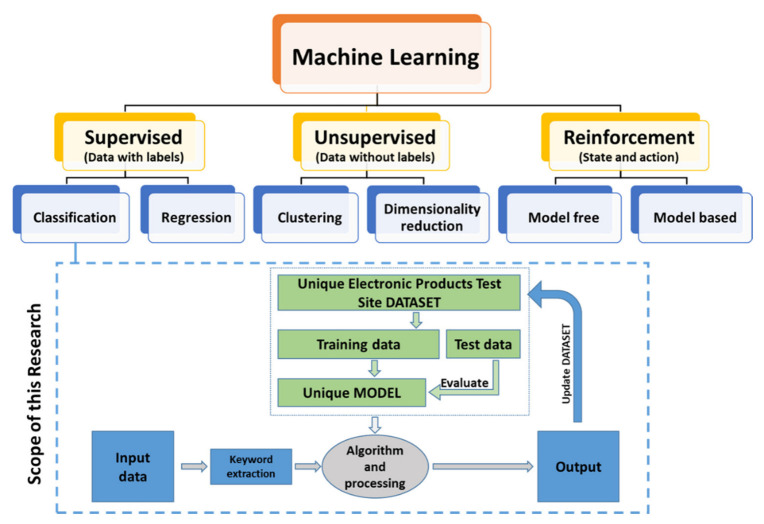
Scope of the machine learning within this research.

The details of the machine learning algorithm are presented in this section. Figure 7 shows the machine-learning scope of this manuscript. This UHF transceiver test site is optimized using a supervised machine-learning algorithm. The machine learning algorithm was developed as part of the research work carried out in [41]. This is a supervised machine learning algorithm where a specific dataset is required. A new dataset was created for this research work. The accuracy of this dataset is dependent on historical data which is collected and performance is shown in terms of reduced test time and improved quality.

**Figure 8:**
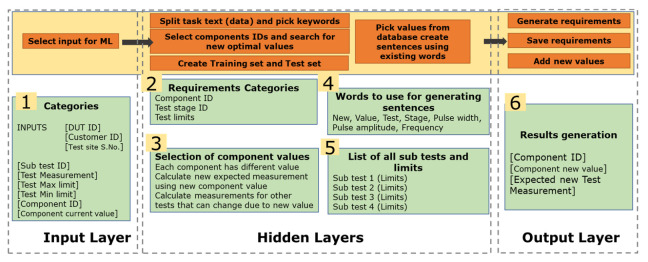
Machine learning algorithm dataset.

In Figure 8, the machine-learning algorithm is presented. The figure shows different layers and steps. The machine learning algorithm takes information through the input layer. This input is automatically acquired when a sub-test fails, and a component value has to be changed. The algorithm then searches through the database and finds a new optimal value. When selecting a new component value, the algorithm also considers previous test results because the results of some other tests can also change when a new component value is used. The algorithm not only checks the new expected measurement value for the sub-test under consideration but also other tests and suggests a value. This is done within the hidden layers. The result is generated through the output layer. A new and unique dataset was created for this research work which is then processed within the hidden layers.

## 6. Implementation and Validation of the UHF Transceiver Test Site

**Figure 9:**
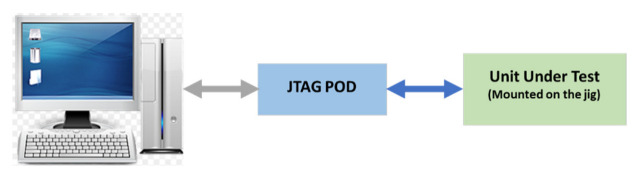
Boundary scan test block diagram.

The UHF transceiver is tested through various test stages. The details of each test stage are presented in this section. The first test stage is the boundary scan test where the UUT goes through an interconnection test to make sure there are no connectivity issues, i.e., the tracks are not shorted nor is the circuit open. The other step is to download the firmware. These tasks are carried out using an onboard JTAG port. Figure 9 shows the block diagram of the UUT interconnections.

**Figure 10:**
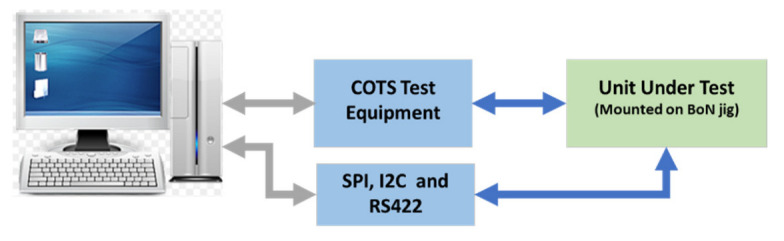
Functional test block diagram.

The next test stage is to test the functionality of the digital control circuit of the UHF transceiver. The UUT can only be tested at this stage if the previous test is successful. During this test, the ADC, DAC, and digital interfaces of the UUT are tested. The test equipment is controlled via the LAN interface. For this test stage, a few USB converters are used. Figure 10 shows the block diagram of this test stage.

**Figure 11:**
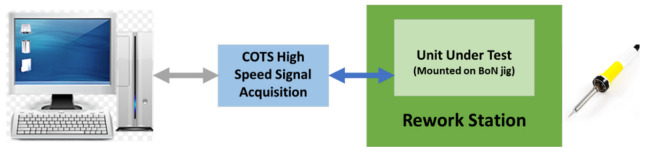
RF transmit circuit test block diagram.

**Figure 12:**
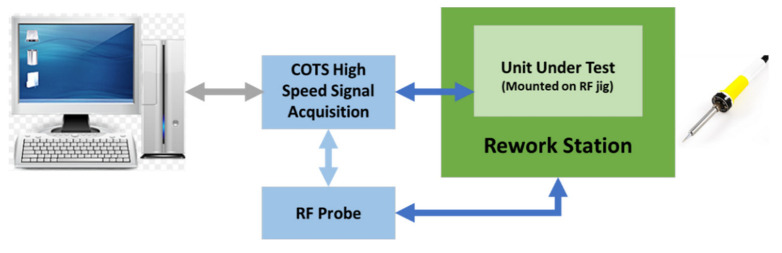
RF tuning circuit and gain test block diagram.

The next two test stages are implemented to test “the receive and transmit” circuit of the UHF transceiver. These are RF tests and require the response of the UUT to be within the operating frequency. The UUT is fitted with default component values which are required to be changed to bring the measurements within the test limits. For this test stage, the test software using machine learning displays a message suggesting a new component value. The test operator can then perform a rework on the PCB, fit the new component, and continue with the test. Depending on the test result, the test operator may have to repeat this stage until the correct component value is fitted. The block diagram of this test stage is shown in Figure 11 and Figure 12.

**Figure 13:**
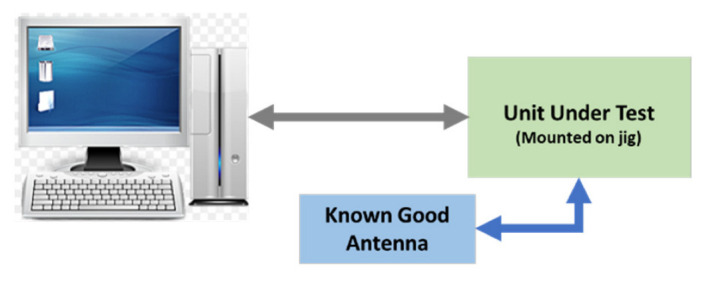
Final test block diagram.

The UUT is tested at the last stage for basic functionality, i.e., if the UHF transceiver can transmit and receive a known signal. This test is performed after fitting the UUT inside the casing. At the end of each test stage, the test results are stored on the test computer. The block diagram is shown in Figure 13.

**Figure 14:**
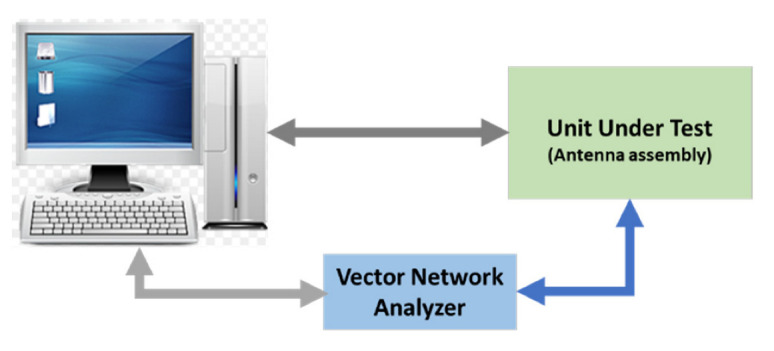
Antenna test site block diagram.

The UHF transceiver antenna is tested using a separate test site before being fitted inside the UUT casing. This is a passive test where the antenna is tested for frequency response and impedance. The block diagram is shown in Figure 14. 

## 7. Discussion and Results

The manuscript presents a process to setup up a test site for a UHF transceiver product. Testing is a vital step in manufacturing which is critical in maintaining and improving product quality. Depending on the time to market and cost, some products do not go through the testing process. This leads to a high risk at the customer end who is receiving the product, and this consequently results in quality issues. The manuscript highlights some of the challenges when setting up test sites for RF electronic products and provides a new technique based on machine learning to improve yield. In the literature, a machine learning algorithm has not yet been employed to solve such types of problem. The optimization of this test site using automation and machine learning leads to increased yield, which results in financial benefit and increased test capacity. 

The test site is setup to test a UHF transceiver. The test site hardware includes test jigs and test equipment to take measurements from the UUT within its operating frequency range. The automated test site is controlled using test software developed in LabVIEW. The UUT is tested during manufacturing through different test stages where the component values are required to be modified to maintain UUT functionality within the operating frequency range. The test site also records all test results, modifications, and other parameters and provides traceability for each UUT tested through this test site.

The first few batches of the UUT test site are tested manually to streamline the test process and finalize the test sequence and the subsequent measurements. This is a tedious process where the operator is required to setup the test equipment manually for each UUT. After taking the measurements the test results were recorded manually and checked against the test limits. Some component values are required to be changed to bring the measurements within the test limits. This is where the test operator is required to make a decision and select a component value. It is observed that sometimes the test operator took a few iterations to select the correct component value. For some measurements, the test operator is required to do some calculations, and based on that needed the test equipment settings are changed. 

The process also highlights inconsistencies and errors. A UUT batch size of 10 is tested through a manual process. The test time for the first UUT batch through the manual test process is 400 min i.e., 40 min per UUT. The next step is to automate the test site using automated test software which controls the test equipment, acquire measurements, and store test results. Another batch of UUTs is then tested through an automated test site and a significant increase in yield is achieved. The test time per UUT after automation is 20 min which means that 20 UUTs are tested in 400 min which shows a 100% increase in yield. 

The next step is to find a solution for reducing the inconsistencies and to further reduce the test time to cope with the UUT quantities. Different scenarios and techniques are considered and at the end of it, machine learning is applied to solve this problem. Different machine learning algorithms are reviewed and evaluated for this purpose. The test software is then upgraded to add machine learning functionality. A new machine learning dataset is created to solve this problem. The third batch of UUT is tested after this upgrade which shows increased consistency and further reduces the test time per UUT. This time a total of 34 UUTs are tested in 408 min which means the test time per UUT is further reduced to 12 min per UUT. The quality is also improved due to increased consistency after the application of machine learning. 

**Figure 15:**
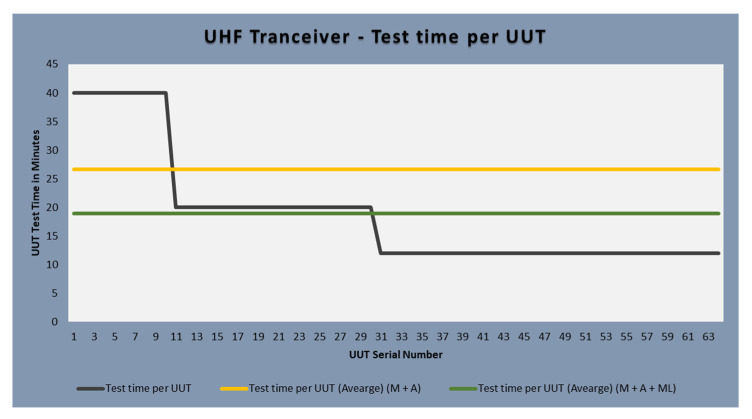
UUT serial number v. test time per UUT.

Figure 15 shows the improvements achieved through automation and machine learning. The initial test time per UUT for manual testing was 40 min per UUT which is now reduced to 12 min, which is a 230% increase in yield. Using machine learning it was possible to increase the yield while improving the quality of the UHF transceiver. 

This represents huge weekly savings, and the weekly yield increased from 35 tests per week to 105 tests per week. This means 23 h were saved per week per operator. The test site is now more reliable, repeatable, and stable. Operator error is reduced, and consistency is increased. 

**Figure 16:**
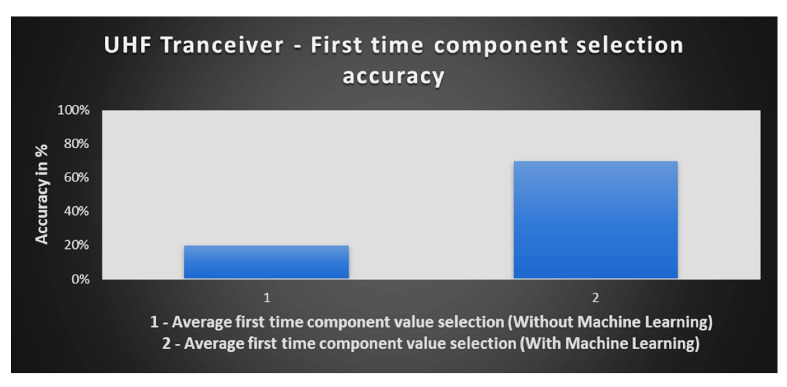
Comparison of first-time component value selection pre and post-machine learning.

Figure 16 presents the improvement after the application of machine learning. The application of a machine learning algorithm reduced the component value selection and rework time which is necessary to tune the UHF transceiver circuit. The accuracy of the selection of component values based on machine learning is expected to increase with the collection of more test data. The accuracy of machine learning is around 70% on average after testing the first batch through machine learning-based software. This means that the correct component value is selected through a machine learning algorithm 7 out of 10 times. At the start, the operator was only able to select the correct component value 2 times out of 10. This means the accuracy is increased from 20% to 70% through machine learning. 

The performance criteria used in this type of industry is the First-Time Yield (FTY) and ultimately the overall yield. The FTY is the ratio of the UUT’s pass the test first time and the total UUTs tested. The UUT’s that fail the test are reworked, repaired and retested. Depending on the process, most of the failed UUTs pass the retest. At this stage, the overall yield is calculated which is the ratio of the UUTs pass the test and the total UUTs tested. Some companies report FTY while others use overall yield as the Key Performance Indicator (KPI).

(1) $$First\;time\;Yield\;\left(Component\;selection\right)=\frac{Correct\;component\;selected\;first\;time}{Total\;comonents\;replaced}$$

The FTY for both the manual and automated test sites where the component replacement needed is 20%. This means that the test operator was only able to select 2 out of 10 correct components first time. The difference between the manual and automated test sites is the reduced test time. After the application of machine learning based algorithm the FTY is increased to 70%.

Replacing components on the PCB can reduce the reliability of the UUT if any track or pad, etc., is damaged during the process. These damaged tracks or pads are then repaired based on the company’s quality control policy. The UUT passing the retest after rework is reliable and ready to be shipped to the customer. We have demonstrated through test results that after the application of machine learning, the first-time component selection is improved which improves UUT integrity, as fewer components need to be replaced.

**Figure 17:**
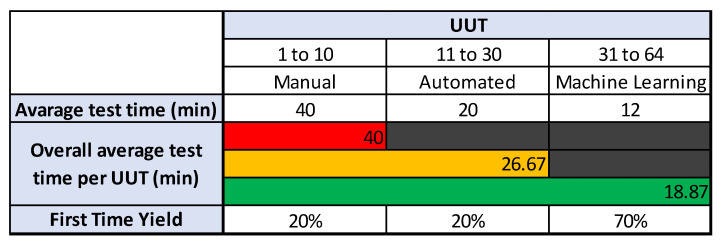
Final test and validation results in tabular form.

Figure 17 presents a comparison of the three batches. The first batch of 10 UUTs is tested using manual process where the average test time per UUT is 40 min and FTY is 20%. The second batch of 20 UUTs is tested after the test site is automated. The average test time is 20 min per UUT and FTY is 20%. The average test time per UUT for the first two batches combined is 26.67 min. Finally, the third column shows the details of last batch of 34 UUT where average test time per UUT is 12 min and FTY is increased to 70%. The overall average test time per UUT for all the batches is 18.87 min. In the figure, red color shows the average test time for manual testing, amber color shows the average test time for the two UUT batches tested manually and after automation, while the green color shows the average test time of the three batches combined.

**Figure 18:**
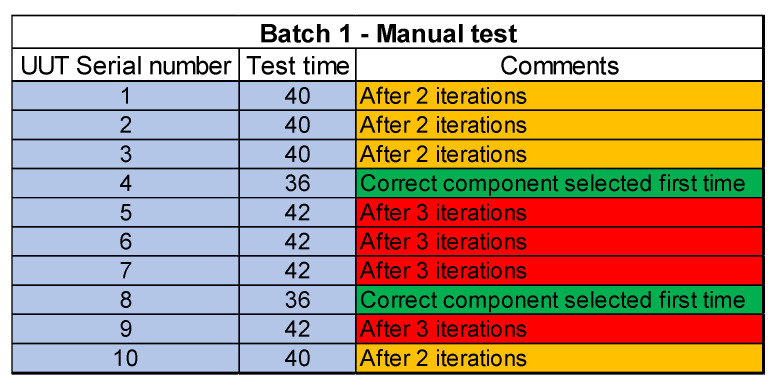
Test results for batch 1- manual test site.

**Figure 19:**
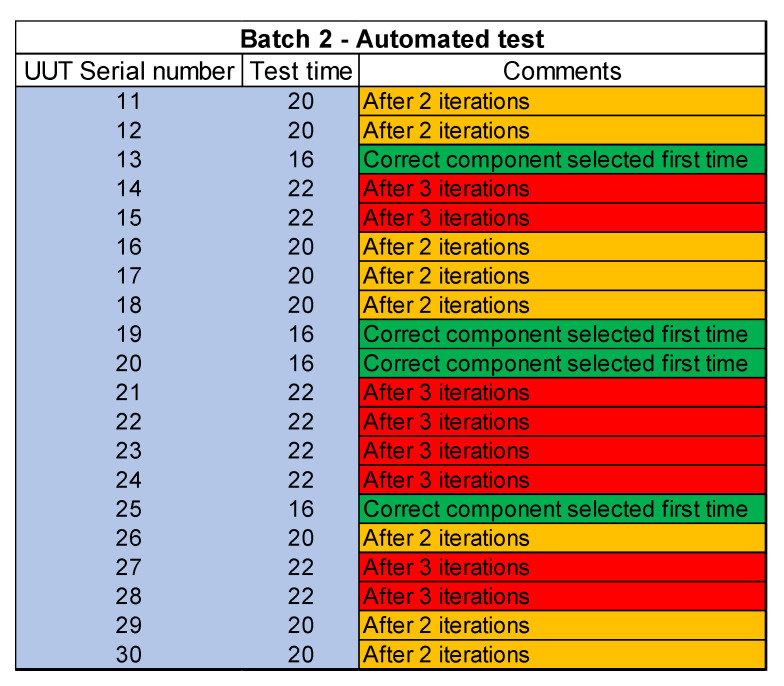
Test results for batch 2- automated test site.

**Figure 20:**
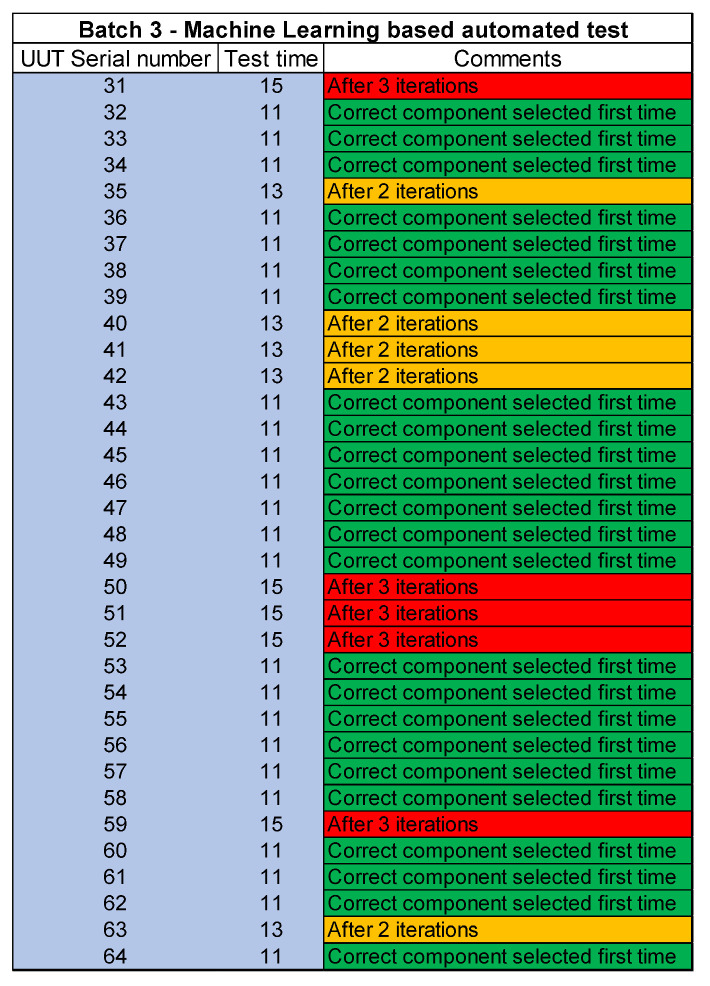
Test results for batch 3- machine learning based automated test site.

Figure 18, Figure 19 and Figure 20 lists the details of the three batches tested. Individual serial numbers are in column 1 with the individual UUT test time in minutes in columns 2. The last column provides details of number of iterations for component change. In the figure red color shows the worst-case scenario where the correct component is fitted after 3 iterations. The UUTs where the correct component is fitted after 2 iterations is highlighted in amber color while the UUTs where the correct component is fitted after first iteration is shown as green.

## 8. Conclusions and Future Work

In this study, machine learning is applied for testing an RF electronic product. The test site was initially setup as a manual site to validate the sequence and measurements. In the next step, the test site is automated which reduced the test time per UUT from 40 min to 20 min. This was a significant improvement in yield but there were still some inconsistencies as the operator was required to select some component values. The processes are sometimes completed only after a few iterations to select the optimum component value. These problems increase test time and reduce test reliability because a component is replaced on the PCBA more than one time. After changing the components, the UUT is retested and a PASS result means the UUT is reliable. At this stage a machine learning algorithm is applied which selected the optimal component value for the operator by processing the historical data, i.e., the component values selected based on measurements taken. This reduced the test time further and increased consistency thus improving the product quality. The final test time is 12 min per UUT. The increase in yield is 100% after automation and another 130% after the application of the machine learning algorithm. The overall yield is increased by 230% which shows how machine learning can benefit in tackling this type of problem. 

More machine learning algorithms can be explored and incorporated to further improve the testing and manufacturing of RF electronic products. Every time a new fault has been entered, the machine learning-based test software will add this to the fault database and will identify this fault next time and suggest an optimal solution to the operator. Test software can also perform analysis for a current batch of UUTs and suggest what the operator can do to resolve an issue.

## Figures and Tables

**Table 1:** RF electronic products reviewed.

S. No.	Type	Specifications
1	Hybrid Digital and RF Equipment	Digitally controlled RF Systems
2	RF cables	High and Low RF cables
3	RF Antennas	Various types
4	Radars	Ground penetrating systems are also included
5	RF Products	3 GHz or less
6	Sat Comm Systems	For Satellite TV
7	Transducers/Sensors	IoT devices, IP-based control
8	Underwater Modems	High and Low speed/power

**Table 2:** Reviewed RF and other test sites.

Device under Test	Software	Hardware
Aerospace [6]	Visual Basic	Harness, Automated
Avionics [28]	Not mentioned	PXI system
Satellite control [7]	Java	ATE, digital interfaces
Digital interfaces, DAQ [11,15,18,24]	LabVIEW	COTS equipment, digital interfaces
Low frequency [19,29]	LabVIEW, Python [30]	Bed of nails, PXI, DAQ
IoT [2,16]	COTS	COT equipment
PCB [5]	LabVIEW	Bed of nails, PXI
Backplane [4]	COTS	COTS equipment
Harness [26]	LabVIEW	DAQ
Antenna [12,13,20]	LabVIEW	Digital control board, COTS equipment
Open layer board [17]	LabVIEW	PXI, COTS equipment
RF probe [27]	LabVIEW	COTS equipment
RF unit [25]	LabVIEW	Digital interface
Software-defined radio [23]	LabVIEW	COTS equipment, digital interfaces
Wireless devices [8,10]	LabVIEW	COTS equipment, Sensors

## Data Availability

Data not available.
